# Selective Effects of Postural Control on Spatial vs. Nonspatial Working Memory: A Functional Near-Infrared Spectral Imaging Study

**DOI:** 10.3389/fnhum.2018.00243

**Published:** 2018-06-13

**Authors:** Yifan Chen, Yanglan Yu, Ruoyu Niu, Ying Liu

**Affiliations:** ^1^School of Kinesiology, Shanghai University of Sport, Shanghai, China; ^2^Key Lab of Cognitive Evaluation and Regulation in Sport, General Administration of Sport, Shanghai, China

**Keywords:** fronto-parietal network, spatial working memory, dual-task paradigm, fNIRS, center of pressure

## Abstract

**Background**: Previous evidence suggests that postural control processing may be more related to spatial working memory (SWM) than to nonspatial working memory (NWM). Methodological discrepancies between spatial and nonspatial cognitive tasks have made direct comparisons between the two systems difficult.

**Methods**: To explore the neural mechanisms of SWM and NWM relative to that of postural control, participants were subjected a cognitive-posture dual-task paradigm, consisting of a 3-back letter working memory (WM) task, using physically identical stimuli with spatial and nonspatial components memorized in different sessions, and a standing balance task with a tandem stance. Additionally, there were two control sessions: a single-postural control session wherein participants pressed mouse buttons at random while standing; and a single-cognitive task control session wherein subjects completed a WM task while seated. The subjects underwent functional near-infrared spectral imaging (fNIRS) during task performance, wherein oxygenated hemoglobin concentration ([HbO]) was measured in frontal and parietal regions.

**Results**: Postural control reduced discernment in the SWM task significantly, but did not affect NWM task performance. fNIRS showed that postural control had a significant tendency to decrease the [HbO] in the frontal-parietal network of the left hemisphere when participants completed the SWM task. No posture-associated differences in [HbO] were observed in NWM-related areas during NWM task performance. Behavioral and fNIRS data demonstrated that postural control had a selective interaction with SWM. Specifically, postural control reduced SWM discrimination and SWM-related brain activity (frontal-parietal network), but not NWM discrimination or NWM-related brain activity. Furthermore, the multiple linear regression analysis showed that SWM, but not NWM, was an important predictor of postural control. These results suggest that postural control may share more cognitive resources with SWM than with NWM.

## Introduction

Postural control refers generally to the maintenance of body-posture and practically to the stability of the body’s center of pressure (COP). In daily life, people maintain a well-balanced posture while standing or walking without conscious monitoring of their posture. Lower components of the central nervous system, including the cerebellum, brain stem and spinal cord, play a major role in the physiological control of standing posture. Therefore, postural control has traditionally been considered an automatic motor task, requiring minimal higher cognitive resources (Sveistrup and Woollacott, 1996). However, researchers have found an interaction between postural control and cognitive task performance, indicating that postural control is not a fully automatic process, but rather may require active cognitive processes (Woollacott and Shumway-Cook, 2002; Yogev-Seligmann et al., 2008), including complex information processing, such as perception, decision-making and motor control (Watson, 1999). Usually, a cognition-posture dual-task paradigm is employed in such studies. When subjects perform a postural control task while also engaging in a cognitive task (e.g., having a conversation, thinking, or decision-making), performance in the cognitive task, postural control task, or both tasks is impaired, relative to that shown during performance of each task alone, indicating that the postural control task involves the use of cognitive resources (Schmidt and Lee, 1999).

Some research has suggested that because postural control has spatial component, cognitive tasks that have spatial processing requirements might create greater dual-task interference (Fuhrman et al., 2015). This suggestion fits well with many behavioral findings on the interaction between spatial working memory (SWM) and postural control. Kerr et al. (1985) first demonstrated this interaction in a human study wherein the subjects’ performance in the Brooks’ spatial/nonspatial memory task of repeating spatial/nonspatial-related sentences was impaired while in a balance-demanding standing position vs. while sitting. The results showed a significant selective decline in accuracy for the spatial memory task during standing postural control (Kerr et al., 1985). Concurrent postural control demands in a challenging stance posture task reduced reaction speed in a spatial memory task, but not in an object memory task (VanderVelde et al., 2005). Maintaining balance in a challenging stance decreased accuracy and increased reaction time (RT) in a spatial task, but not in a nonspatial task under dual-task conditions compared with each single cognitive task condition (Chong et al., 2010). One potential explanation for this behavioral interaction is that postural control and cognitive tasks may involve processing, particularly spatial cognitive processing, in common brain regions (Fraizer and Mitra, 2008). However, there has been relatively little research attempting to provide direct verification of a common neural basis for postural control and cognitive processing.

Potentially, common neural mechanisms may be derived from the respective neural mechanisms of posture and cognitive processing, especially from overlaps between spatial cognitive processing and posture control. Neuropsychological studies have demonstrated that the frontal-parietal network plays an important role in the processing of both SWM and nonspatial working memory (NWM; Klingberg, 2006; Ricciardi et al., 2006; de Souza Custódio et al., 2013). Meanwhile, the frontal cortex and parietal cortex have been suggested to be neural candidates for postural control In an event-related potential study, Little and Woollacott (2015) found a postural control-evoked N1 component in a motor cortical area. The authors of some functional near-infrared spectroscopy (fNIRS) studies have suggested that the prefrontal cortex (PFC) may be essential for balance control (Suzuki et al., 2008; Caliandro et al., 2012, 2014). Therefore, the frontal-parietal network may provide neural underpinnings for both postural control and working memory (WM) processes.

More importantly, the spatial characteristics of postural control suggest neural overlap between SWM and posture control. It has been proposed that different segments of the PFC may be responsible for different types of WM, with the dorsal region being characterized as necessary for the storage of spatial information and the more ventral region being characterized as necessary for processing of non-spatial information (anatomical evidence, Cavada and Goldman-Rakic, 1989; Romanski et al., 1999); physiology evidence (Wilson et al., 1993; Diwadkar et al., 2000; D’Esposito et al., 2000; Walter et al., 2003; Meyer et al., 2011). Based on some fNIRS study findings, Mihara et al. (2008) suggested that the bilateral dorsolateral PFC and frontal eye field play important roles in the maintenance of standing balance. Such findings are consistent with the possibility of there being substantial interference between spatial information processing and postural control processes due to a sharing of dorsolateral PFC neural resources.

At present, a relationship between posture control and SWM has been demonstrated only at the behavioral level. An interaction between SWM and postural control has yet to be demonstrated at the neural level. Methodologically, previous research has used incongruous SWM and NWM tasks. For instance, VanderVelde et al. (2005) used dot stimuli in their SWM task and an irregular graph in their NWM task. Chong et al. (2010) used the Retro-7 task to test SWM and a word-generation task to test NWM. The inherent differences from physically non-identical stimuli between these pairs of tests make direct comparisons between SWM and NWM systems difficult.

To facilitate comparison between SWM and NWM results, we used the 3-back task, a widely used WM task, with physically identical stimuli across SWM and NWM components. The core aim of this study was thus to explore the complex interaction between WM (spatial/nonspatial) and postural control. A dual-task paradigm was used to explore interactions between WM and postural control. We also employed fNIRS, a noninvasive neuroimaging method that uses a tissue’s absorption of near-infrared light to measure relative oxygenated and deoxygenated hemoglobin concentration (Chen et al., 2015), and is not restricted to a limited testing environment (Cui et al., 2015), making it suitable for research involving actively moving subjects (Kovelman et al., 2008). Our analysis of hemodynamics targeted frontal and parietal areas because previous research has suggested that WM and postural control may interact in these areas.

## Materials and Methods

### Subjects

Eighteen right-handed volunteers with normal or corrected-to-normal vision, a normal body mass index (18.5–23.9), and proficiency with English as a second language participated in the study. Data from one female subject were discarded due to an excessively low correct percentage (<80%) in the cognitive tasks. Data from the remaining 17 subjects were included in the final analyses. The final cohort included eight men and nine women, with a mean [± standard error (SE)] age of 22.47 ± 0.63 years (range, 19–26 years), a mean height of 169.18 ± 2.25 cm (range, 150–180 cm), and a mean weight of 60.25 ± 2.28 kg (range, 45–75 kg). Subjects provided written informed consent and were paid for their participation. The study followed the ethical guidelines of the Declaration of Helsinki and was approved by the local ethics committee at Shanghai University of Sport, Shanghai, China.

### WM Task

#### Stimuli

Stimuli were presented on a computer screen (resolution of 1920 × 1080 pixels and refresh rate of 60 Hz) positioned 1 m from the eyes of the subject. Stimulus presentation was controlled by the Psychtoolbox package for Matlab (Brainard, 1997; Pelli, 1997). All displays had a black background. The fixation mark at the center of the screen was a white cross with a degree of visual angle (dva) of 0.8°. Eight pairs of uppercase and lowercase English letters (A, a, B, b, D, d, E, e, F, f, G, g, H, h, J, j) constituted the target stimulus pool. One certain letter was chosen based on the dissimilarity between the uppercase and lowercase of that letter. The target was one letter in the “pool”. Two imaginary circles were 0.68° and 2.5° dva from the fixation point, respectively. Four imaginary lines also went through the center of the screen, one horizontal, one vertical and two mutually perpendicular lines (45° in the horizontal direction) composed those lines. The two imaginary circles intersecting those four imaginary lines determined 16 possible positions on screen. The target was white with a 0.8° dva.

#### Task and Procedure

**Figure 1:**
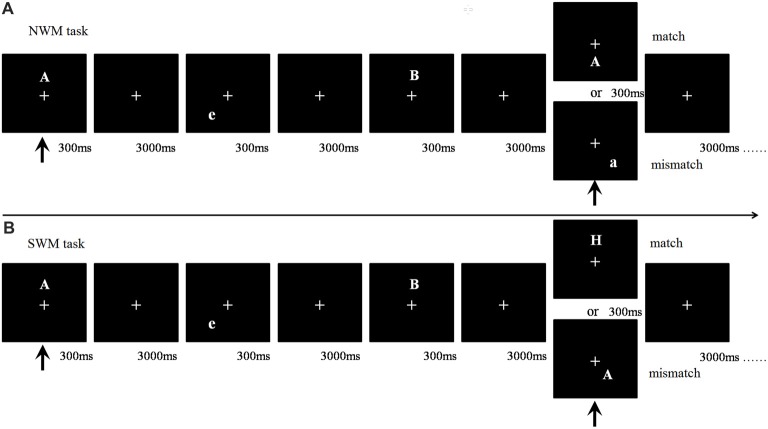
Flow chart of working memory (WM) task types. **(A)** Nonspatial working memory (NWM) task. The first arrow indicates the appearance of the first (target) stimulus and the beginning of the task. The second arrow indicates the appearance of the fourth (probe) stimulus, where participants were required to match the fourth and first stimuli. A match trial occurred if the 4th and 1st letters matched (case sensitive), otherwise it was a mismatch trial. **(B)** Spatial working memory (SWM) task. Participants needed to match the location of a letter between the 4th and 1st trials. A match trial occurred if the 4th and 1st letters had matching locations otherwise it was a mismatch trial.

The fixation cross was presented on the screen throughout each trial, with a maximum trial duration of 3 s. Within each trial, a randomly selected letter served as the target stimulus and was displayed in a randomly selected position for 300 ms, as shown in Figure 1. Subsequently, in each of two WM interference phases, a different letter was shown in a different location also for 300 ms, following a 3000-ms no stimulus interval. Finally, a probe stimulus letter was presented for the subject to judge as a match or mismatch. The ratio of matching stimuli to mismatching stimuli was 1:2.

Subjects were instructed to press a match or mismatch button to register their judgment, and then proceed immediately to the next trial. The left and right mouse buttons, to be pressed with the right index and middle finger, respectively, served as the judgment registration method, with button-response pairing counter-balanced across subjects. Subjects were instructed to answer as quickly and accurately as possible.

The WM task included SWM and NWM components. In the SWM component, participants were asked to match the stimulus’ location match between the target letter and the probe letter within each 3-back sequence. In the NWM component, participants were asked to match the stimulus’ letter identity (case sensitive) between the target letter and the probe letter within each 3-back sequence.

### Postural Control Task

**Figure 2:**
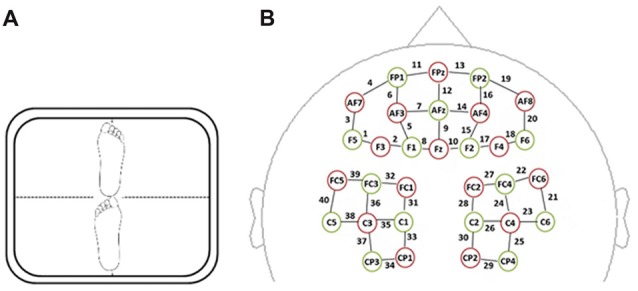
**(A)** Tandem barefoot standing postural control task scheme. The side of the heel of one foot was touching the big toe of the other. **(B)** Spatial profile of functional near-infrared spectral imaging (fNIRS) probes. The red circles indicate the 16 optical sources, the green circles indicate the 15 detectors, and the black numbers (1–40) indicate fNIRS channels. The optical sources and detectors were positioned on the international 10–20 standard positions.

We adopted the tandem stance paradigm as our postural control task because it is a challenging postural control task that has been used in previous studies to explore the interaction between cognitive performance and postural control (VanderVelde et al., 2005; VanderVelde and Woollacott, 2008). Postural stability was measured by tracking ground reaction forces during tandem standing. The time series data of the COP trajectory was recorded via Super Balance (ACMEWAY, Beijing, China) as shown in Figure 2A. Data were sampled at a frequency of 100 Hz. The whole path length (WPL), envelope area (ENV) and mean speed (MS) of the COP trajectory were calculated as metrics of postural control.

### Dual-Task Paradigm With Neuroimaging

To investigate the interaction between WM and postural control, participants completed the WM and postural control tasks at the same time, as a dual-task session. They stood barefoot on the standing area of a balancer during tandem standing, balancing while being presented with WM task visual stimuli in front of them and responding to the WM trials. Participants held a wireless mouse for WM trial response registration in their dominant hand while allowing their arms to hang at their sides. There was also one ~65-s resting session wherein subjects sat quietly and watched a black screen as well as two kinds of single-task sessions: (1) a single-postural control session wherein participants pressed mouse buttons at random while standing; and (2) a single-cognitive task session wherein subjects completed the WM task while seated. Neuroimaging data were recorded while patients completed all of the sessions.

**Figure 3:**
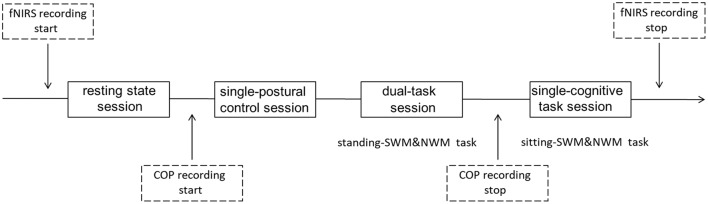
Schema of current design. All subjects had a resting state session for ~65 s at the beginning of the testing period. Then, half of the subjects had a single-postural control session before the dual-task and single-cognitive task sessions, while others completed the single-postural control session last; the session type order was counterbalanced across subjects. Different WM tasks were also counterbalanced within subjects. Each WM-related task had two 65-s blocks. fNIRS data were collected throughout all sessions. Center of pressure (COP) data were recorded during the single-postural control and dual-task sessions.

SWM and NWM were tested in different blocks, as were dual-task and single-task paradigms, to enable dissociation of the cognitive demands. Hence, the following behavioral task blocks were analyzed: standing-SWM, standing-NWM, sitting-SWM and sitting-NWM. The subjects completed a dual-task session consisting of the standing-SWM and standing-NWM blocks (2 blocks each), a single-cognitive task session consisting of the sitting-SWM and sitting-NWM blocks (2 blocks each), a single-postural control session (1 block), and a resting state session (1 block), constituting 10 blocks in all. The order of these different experimental condition sessions was randomized across the subjects, with a 3-min break between successive sessions (Figure 3). Each WM-related block was composed of 27 trials and each block lasted ~65 s. The whole experiment lasted ~30 min. One day before the experiment, participants were invited to laboratory to preview the experimental setting. To minimize the effect of practice, participants completed a 30-min training/practice session the day before the experiment.

### Hemodynamic Data Acquisition

A multi-channel, continuous wave, fNIRS instrument (NIRScout, NIRx Medical Technologies LLC, Minneapolis, MN, USA) was used to monitor hemodynamic activity during task performance and in a resting state. The sampling rate was 3.91 Hz. The fNIRS probe consisted of 16 dual-wave length sources (780 nm and 830 nm) and 15 optical detectors, which covered both the frontal and parietal areas bilaterally. The distance between an emitter and a detector was 3 cm. One emitter and one detector formed a channel. Forty channels were formed, 20 of which were distributed in the frontal area, with the remaining 20 being distributed over parietal areas (Figure 2B). The correspondence between fNIRS channel locations and specific brain regions was from studies of Okamoto et al. (2004, 2009) and Tsuzuki et al. (2007). The probes were arranged according to a 10/20 electroencephalogram system with some adjustments to ensure that each emitter was 3 cm away from its corresponding detector.

### Data Analysis

#### Behavior

To minimize the impact of RT outliers, trials with RTs <150 ms or >2000 ms were discarded. In addition, a trimming procedure was applied to discard outliers falling outside three standard deviations around the mean. In accordance with signal detection theory (Goodenough et al., 1972), discernment (d′) and reaction tendency (*β*) WM variables were calculated under various experimental conditions in Matlab software (MathWorks Inc., Natick, MA, USA). The effects of the experimental conditions on match accuracy, RT (trimmed mean), d′ and *β* were determined by multivariate analysis of variances (ANOVAs) in SPSS 20.0 software (SPSS Inc., Chicago, IL, USA). If any main effect or interaction existed, paired sample *t*-tests were used to make further comparisons. Mean values for the behavioral variables were reported with SEs.

#### Hemodynamic Imaging

Optical data were converted into hemoglobin signals with arbitrary units in accordance with the modified Beer-Lambert Law (Cope et al., 1988). Because oxygenated hemoglobin signals have a better signal-to-noise ratio than deoxygenated hemoglobin signals (Niu et al., 2013; Schaeffer et al., 2014), only oxygenated hemoglobin concentration ([HbO]) data were used. The [HbO] data were analyzed in nirsLAB software (Xu et al., 2014). After removing discontinuous shifts from the data time series, [HbO] signals were bandpass filtered between 0.01 Hz and 0.2 Hz to remove baseline drift and physiological noise (e.g., heartbeats). Then, each participant’s [HbO] in each session was calculated. Hemodynamic data were then baseline corrected based on the mean value of all signals from each block (5 s before to 15 s after the block). The [HbO] data were then averaged across subjects.

We defined region of interest (ROI) channels as those channels with maximal [HbO]. After averaging [HbO] across participants, mean [HbO] in the single-cognitive task sessions (including the sitting-SWM task and sitting-NWM task) was subtracted from mean [HbO] in the resting state session. Then, the mean difference values for each channel between the single-cognitive task and resting state sessions were arranged according to descending magnitude, and the top 15% of channels (greatest values) were defined as channels of interest. The multi-channel fNIRS space was converted into traditional Montreal Neurological Institute space (Cutini et al., 2011); based on their spatial distribution relative to the Anatomical Automatic Labeling template, those channels of interest were associated with ROIs (three ROIs for the SWM task and two ROIs for the NWM task) as shown in Table 1. Subsequently, averaged [HbO]s across the channels within each ROI were analyzed. One-way repeated measures ANOVAs for the measure averaged [HbO]s with ROIs as a factor were conducted to determine ROI with the maximal activity, separately for SWM and NWM. *Post hoc* analysis (Least Significant Difference) was used to make further comparisons. The effects of the experimental conditions on averaged [HbO]s within ROI with maximal activity were determined by ANOVAs. If any main effect or interaction existed, paired sample *t*-tests were used to make further comparisons. Mean values for [HbO]s are reported with SEs.

**Table 1:** Spatial working memory (SWM)- and nonspatial working memory (NWM)-related regions of interest (ROIs).

Task	ROI	Channels	Hemisphere	Location
	1	1	L	Frontal_Inf_Tri\Frontal_Mid
SWM	2	31 33 36 37	L	Frontal_Mid\Postcentral\Precentral\Parietal_Inf\Parietal_Sup
	3	26	R	Precentral
NWM	4	37 40	L	Frontal_Inf_Oper\Parietal_Inf\Postcentral
	5	21 22 24 26	R	Frontal_Inf_Oper\Frontal_Mid\Postcentral\Precentral

*L, left; R, right; Frontal_Inf_Oper, inferior frontal gyrus, opercular part; Frontal_Inf_Tri, inferior frontal gyrus, triangular part; Frontal_Mid, middle frontal gyrus; Parietal_Inf, inferior parietal gyrus; Parietal_Sup, superior parietal gyrus; Postcentral, postcentral gyrus; Precentral, precentral gyrus*.

## Results

### Behavior

#### Postural Control Significantly Reduced SWM Discernment

**Figure 4:**
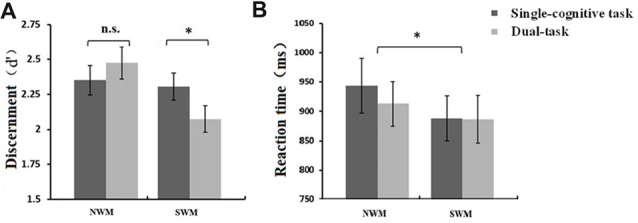
**(A)** Mean discernment, d′, (±SE) of SWM and NWM tasks under different posture conditions. Discernment for SWM in the dual-task session was significantly lower than that in the single-cognitive task session. **(B)** Mean reaction time (RT; ±SE) of SWM and NSW tasks under different posture conditions. RT for NWM task performance was significantly longer than that for SWM task performance. *p < 0.05, n.s., *p* > 0.10.

Our 2 (WM type: SWM vs. NWM) × 2 (posture condition: standing vs. sitting) ANOVAs for d′ and *β* in the SWM and NWM task components revealed a significant main effect of WM type (*F*_(1,16)_ = 4.76, *p* = 0.044) on d′, and an interaction between WM type and posture condition (*F*_(1,16)_ = 5.16, *p* = 0.037). Mean d′ was significantly lower for the SWM task component (2.19 ± 0.10) than for the NWM task component (2.41 ± 0.11). As shown in Figure 4A, paired *t*-tests showed a significant difference in d′ between dual-task and single-cognitive task sessions for SWM (*t* = 2.56, *df* = 16, *p* = 0.022; standing-SWM, mean d′ = 2.07 ± 0.10; sitting-SWM, mean d′ = 2.31 ± 0.10), but not for NWM (*t* = 0.88, *df* = 16, *p* = 0.39; standing-NWM, mean d′ = 2.47 ± 0.11; sitting-NWM, mean d′ = 2.35 ± 0.12). No significant effects of WM type or posture on *β* were found (all *p* > 0.05), indicating that the tendency to respond to trials was independent of these conditions.

Our 2 (SWM vs. NWM) × 2 (standing vs. sitting) ANOVAs revealed a significant main effect of WM type on RT (*F*_(1,16)_ = 5.69, *p* = 0.030), but not accuracy (*F*_(1,16)_ = 1.56, *p* = 0.23). As shown in Figure 4B, mean RT was greater for NWM (927.98 ± 42.06 ms) than for SWM (887.17 ± 38.87 ms). Meanwhile, there was no posture-related effects on RT (main effect of posture: *F*_(1,16)_ = 0.68, *p* = 0.42; interaction: *F*_(1,16)_ = 0.72, *p* = 0.41) or accuracy (main effect of posture: *F*_(1,16)_ = 1.48, *p* = 0.24; interaction: *F*_(1,16)_ = 1.34, *p* = 0.26).

#### Baseline Cognitive Ability Level Related Negatively With a Behavioral Experiment Effect

To determine if baseline cognitive ability level may explain some of the variability in our results, d′ in the sitting-SWM task was considered a baseline cognitive metric. We calculated the difference in d′ between the dual-task session and single-cognitive task session (i.e., a behavioral experiment effect), for both SWM and NWM. A correlation analysis between baseline and experiment effect results showed that there was a significant negative correlation between them for both SWM (*r* = −0.52, *p* = 0.032, *N* = 17) and NWM (*r* = −0.50, *p* = 0.039, *N* = 17; Spearman correlation coefficients, two-tailed tests).

#### SWM Task Tended to Reduce Posture Stability

Paired *t*-tests on WPL, ENV and MS in dual-task vs. single-postural control session suggested a trend toward greater mean WPL in the standing-SWM (1741.69 ± 84.84 mm), but not standing-NWM (1665.58 ± 71.76 mm), blocks relative to that in single-postural control session (1622.26 ± 93.10 mm; *t* = 2.06, *df* = 16, *p* = 0.056). Likewise, there was a trend toward a faster MS in the standing-SWM (24.29 ± 1.17 mm/s), but not standing-NWM (23.23 ± 1.00 mm/s), blocks relative to that in single-postural control session (22.62 ± 1.30 mm/s; *t* = 2.08, *df* = 16, *p* = 0.054). The ENVs for standing-SWM (791.78 ± 100.63 mm^2^) and standing-NWM (713.38 ± 131.37 mm^2^) blocks were similar to the ENV in single-postural control session (808.51 ± 121.87 mm^2^, *p* = 0.90 and *p* = 0.36, respectively).

#### SWM Had a Tendency to Be a More Important Predictor of Postural Control Than NWM

To further explore the relationship between different types of WM and postural control, we conducted a multiple linear regression analysis in which postural control performance was used as the dependent variable, while SWM and NWM performance were predictor variables. The d′ values for sitting-SWM and sitting-NWM tasks were used as measures of SWM and NWM performance, while WPL and MS of COP in the single-postural control session were used as measures of postural control performance. The results showed that the d′ of SWM, but not of NWM, was an important predictor of WPL of COP (SWM: *β* = 431.90, *p* = 0.087; NWM: *β* = 25.03, *p* = 0.91), though only a trend was obtained, likely due to limitations of the small sample size. Similarly, the d′ of SWM, but not NWM, showed a trend toward being an important predictor of MS of COP (SWM: *β* = 6.08, *p* = 0.084; NWM: *β* = 0.33, *p* = 0.92).

### fNIRS

#### Different Types of WM Interacted Differently With Postural Control: Postural Control Tended to Decrease SWM, but Not NWM, Activity in SWM-Related ROIs, Without a Committed Impact on NWM-Related ROIs

A one-way repeated measures ANOVA revealed a main effect of ROI (ROI-1 vs. −2 vs. −3; see Table 1 for definition of areas within each ROI) on mean [HbO] during SWM task performance (*F*_(2,15)_ = 3.80, *p* = 0.027). The mean [HbO] for ROI-2 was maximal (ROI-2, 1.95 × 10^−4^ ± 7.01 × 10^−5^ mmol/L; ROI-3, 1.06 × 10^−4^ ± 7.57 × 10^−5^ mmol/L; ROI-1, 1.78 × 10^−5^ ± 5.93 × 10^−5^ mmol/L). *Post hoc* analysis (Least Significant Difference) showed that the mean [HbO] for ROI-2 was significantly larger than that for ROI-1 (*p* = 0.008). No significant difference was found between the mean [HbO] for ROI-2 and ROI-3 (*p* = 0.13), and for ROI-3 and ROI-1 (*p* = 0.27). The subsequent statistical analysis was conducted for ROI-2 as the region with the maximum mean [HbO].

**Figure 5:**
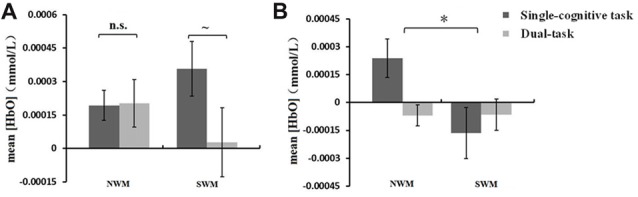
**(A)** Mean oxygenated hemoglobin concentration ([HbO]) (±SE) in region of interest (ROI)-2 during SWM and NWM task performance under different posture conditions. The mean [HbO] for SWM showed a nonsignificant trend toward being lower in dual-task session (standing) than that in single-cognitive task session (sitting). **(B)** Mean [HbO] (±SE) in ROI-5 during SWM and NSW task performance under different posture conditions. The mean [HbO] for NWM was significantly greater than that for SWM. *p < 0.05, ~*p* < 0.08, n.s., *p* > 0.10.

A 2 (SWM vs. NWM) × 2 (standing vs. sitting) ANOVA conducted on mean [HbO] in ROI-2 revealed an interaction between WM type and posture condition (*F*_(1,16)_ = 4.73, *p* = 0.045). As shown in Figures 5A, a paired *t*-test showed a marginally significant effect of posture condition on mean [HbO] for SWM (*t* = 1.87, *df* = 16, *p* = 0.079; standing-SWM, 2.81 × 10^−4^ ± 1.55 × 10^−4^ mmol/L; sitting-SWM, 3.57 × 10^−4^ ± 1.22 × 10^−4^ mmol/L), but not NWM (*t* = −0.081, *df* = 16, *p* = 0.94; standing-NWM, 2.03 × 10^−4^ ± 1.07 × 10^−4^ mmol/L; sitting-NWM, 1.94 × 10^−4^ ± 6.68 × 10^−5^ mmol/L).

A one-way repeated measures ANOVA of mean [HbO] revealed a main effect of ROI (ROI-4 vs. ROI-5; see Table 1 for definition of areas within each ROI) on [HbO] during NWM task performance (*F*_(1,16)_ = 11.19, *p* = 0.004). Because the mean [HbO] for ROI-5 was significantly higher than that for ROI-4, the subsequent statistical analysis was conducted for ROI-5 as the region with the maximum mean [HbO].

A 2 (SWM vs. NWM) × 2 (standing vs. sitting) ANOVA of mean [HbO] in ROI-5 revealed a significant main effect of WM type on [HbO] (*F*_(1,16)_ = 5.01, *p* = 0.040). As shown in Figure 5B, the mean [HbO] of ROI-5 was significantly larger for NWM (8.44 × 10^−4^ ± 6.41 × 10^−5^ mmol/L) than for SWM (−1.15 × 10^−4^ ± 8 × 10^−5^ mmol/L).

#### Baseline Cognitive Ability Level Related Negatively With a Neural Experiment Effect

We used mean [HbO] in the region with the maximum mean [HbO] in single-cognitive task sessions (SWM: ROI-2; NWM: ROI-5) as the subjects’ cognitive ability baseline, and calculated the difference in mean [HbO] between the dual-task and single-cognitive task sessions (i.e., a neural experiment effect) in the corresponding ROI for both SWM and NWM. Correlation analysis showed that there was a significant negative correlation between cognitive ability baseline and the neural experiment effect for both SWM (*r* = −0.61, *p* = 0.009, *N* = 17) and NWM (*r* = −0.80, *p* < 0.01, *N* = 17; Spearman correlation coefficients, two-tailed tests).

#### Behavioral Data Related Positively With fNIRS Data in SWM-Related Tasks

**Figure 6:**
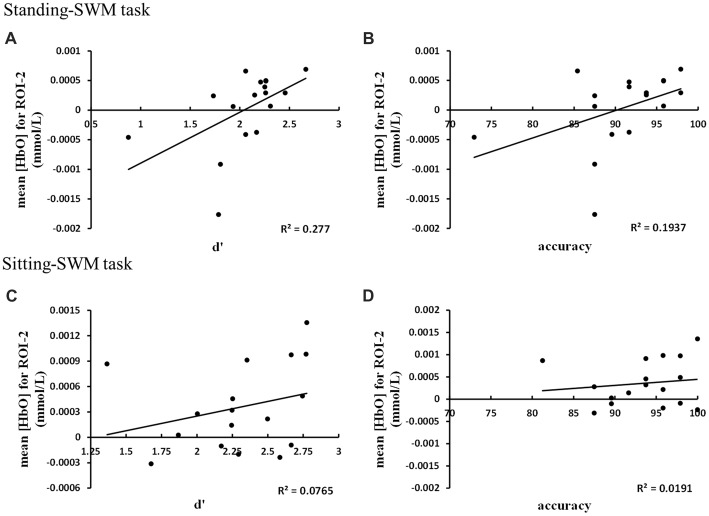
**(A)** The mean [HbO] for ROI-2 correlated positively with d′ in the standing-SWM task. **(B)** The mean [HbO] for ROI-2 correlated positively with accuracy in the standing-SWM task. **(C)** The mean [HbO] for ROI-2 had a marginally significant positive correlation with d′ in the sitting-SWM task. **(D)** The mean [HbO] for ROI-2 did not correlate with accuracy in the sitting-SWM task.

Correlation analysis between behavioral data and hemodynamic response in the regions with the highest mean [HbO] values (Figure 6) showed that the mean [HbO] for ROI-2 correlated positively with d′ and accuracy in the standing-SWM task (d′: *r* = 0.68, *p* = 0.003, *N* = 17; accuracy: *r* = 0.53, *p* = 0.03, *N* = 17). In the sitting-SWM task, the mean [HbO] for ROI-2 had a marginally significant positive correlation with d′(*r* = 0.46, *p* = 0.066, *N* = 17), but did not correlate with accuracy (*r* = 0.21, *p* = 0.41, *N* = 17). For ROI-5, the behavioral data did not correlate with the mean [HbO] in either the standing-or sitting-NWM task (all *p* > 0.20).

## Discussion

The current study employed a two-component 3-back WM task in combination with a tandem stance task, forming a dual-task paradigm, to explore the complex interaction between WM (spatial and nonspatial) and postural control. The SWM and NWM tasks used physically identical stimuli, with subjects expected to be attentive to spatial position in the former and letter identity in the latter. Behavioral data showed that reaction sensitivity, as measured by discernment in the standing-SWM task was significantly lower than that in the sitting-SWM task, but reaction sensitivity was similar across posture conditions for the NWM experimental conditions. Posture-related data revealed that the subjects had rather unstable posture during the standing-SWM task, compared to that in the single-postural control session. Similar effects were not found in the standing-NWM task. The fNIRS data showed lower [HbO]s in SWM-related areas of the frontal-parietal cortex (ROI-2, see Table 1) during the standing-SWM task than during the sitting-SWM task. A similar effect was not found in NWM-related activity. Hence, postural control demands appear to have more influence on SWM than NWM.

Based on previous evidence summarized in the “Introduction” section, we have inferred that spatial information processing may interfere with postural control. In current study, performance in postural control task, as reflected by WPL and MS, tended to be hindered during the standing-SWM task relative to that in single-postural control session. WPL and MS are important indices of stability (Raymakers et al., 2005; Fraizer and Mitra, 2008). WPL reflects the total length of the COP trajectory throughout the task performance period. The longer the length is, the stronger the balance correcting swings were, reflecting less behavioral stability. MS reflects the speed of COP movement, such that a faster speed reflects worse stability. Hence increased WPL and MS values in the standing-SWM task indicated reduced stability and postural control selectively during SWM task performance (see also, Dault et al., 2001; VanderVelde and Woollacott, 2008). In addition, the multiple regression analyses indicated that SWM predicted postural control more accurately than did NWM, leading to the inference that SWM has a greater effect than NWM on postural control, perhaps due to postural control sharing more processing resources with SWM than with NWM.

Notably, the interaction between postural control and WM appears to be double-sided. Behaviorally, reaction sensitivity in the cognitive task, as reflected by d′, was hindered during the standing-SWM task relative to that in the single-cognitive task session. We did not observe an effect of experimental WM condition on *β*, a measure of the tendency to react, indicating that the task conditions did not affect the subjects’ decision-making *per se*. Additionally, we found that baseline WM performance correlated negatively with the experiment effect, such that a higher WM baseline was associated with less interference between posture control and WM.

On the other hand, we used fNIRS to explore the complex interaction between WM (spatial/nonspatial) and postural control on a neural level. Neural activity in the brain results in increased glucose and oxygen consumption from local capillary beds. The increased cerebral blood flow carries oxygen to active areas where it tends to exceed local neuronal oxygen utilization temporarily, resulting in an overabundance of cerebral blood oxygenation in the active areas, and thus an index of neural activity (Irani et al., 2007). Our fNIRS data showed localized [HbO] increases, and thus activity, in ROI-2 and ROI-5 during performance of SWM and NWM tasks, respectively (see Table 1 for list of areas within each ROI). SWM and NWM tasks both activated the middle frontal gyrus (Brodmann 9) in the dorsolateral PFC, while the NWM task also activated the inferior frontal gyrus (opercular part, Brodmann 44) in the ventrolateral PFC, indicating that the processing of nonspatial information require a more ventral region. These distributions of active areas during WM task performance are in line with previous studies (Wilson et al., 1993; Courtney et al., 1998; Diwadkar et al., 2000; D’Esposito et al., 2000; Finke et al., 2006; Klingberg, 2006; Ricciardi et al., 2006; Meyer et al., 2011; de Souza Custódio et al., 2013; Salallonch et al., 2015). After comparing with the specific areas included in ROI-2 and ROI-5, we found that the SWM task was related to activation in parietal regions, including the inferior parietal lobe and superior parietal lobe. Some studies have demonstrated that the parietal lobe was the primary area of spatial perception (Weale, 1983) and direct spatial movement (Snyder et al., 1997; Milner and Goodale, 2006). The superior parietal lobe plays an important role in spatial selection in visuomotor tasks (e.g., GO/NOGO task; Shibata and Ioannides, 2001), and was associated with deficits on tests involving the manipulation and rearrangement of information in WM (Koenigs et al., 2009). In addition, some studies have shown that parietal lobe function was critical for the control of force and posture, and for the formation of one’s body image and its relation to external space (the guidance of movements, including the eyes, to external objects; Freund, 2003). The parietal lobe is thought to be involved in monitoring posture and body movement of the body (Cooke et al., 2000). People with impaired parietal lobe functions have motor disorders affecting standing up and bodily movements (Murayama et al., 2004), consistent with the notion that the parietal lobe is necessary for posture maintenance. Based on these findings, we suspected that SWM and postural control may share more brain regions, and, interestingly, the fNIRS results confirmed our hypothesis.

The current results showed that the mean [HbO] in ROI-2 during the standing-SWM task was significantly lower than that in the sitting-SWM task. Our findings of different neural activity levels in ROI-2 selectively during SWM task performance between dual-task and single-cognitive task sessions may be consequent to common resource consumption between spatial information processing and postural control. The lack of such a finding in ROI-5 during NWM task memory performance suggests that NWM may be less demanding of resources shared with postural control activity than SWM. In addition, greater baseline mean [HbO] in SWM-related and NWM-related regions was associated with less interference by postural control.

Correlation analysis between behavioral data and hemodynamic responses showed that the mean [HbO] for SWM-related regions correlated strongly with d′ and accuracy in the standing-SWM task, and correlated weakly with d′ in the sitting-SWM task. If changes in oxygen consumption and cerebral blood flow in the brain are specific to WM tasks, one may assume that hemodynamic responses are related to behavior (Schroeter et al., 2002). We did not observe a significant correlation between behavioral and NWM-related regions, perhaps due to the small sample size of only 17 subjects.

Beyond our expectations, we also found some behavioral and neurological discrepancies between SWM and NWM in our study. NWM may require more overall resources than SWM. Behaviorally, RT was significantly longer in NWM tasks than in SWM tasks. Neurologically, mean [HbO] in region with the maximum activity for NWM during NWM tasks was significantly larger than that during SWM tasks while mean [HbO] in region with the maximum activity for SWM during SWM tasks was similar to NWM tasks, suggesting, at least tentatively, that NWM task may require more brain resources. This explanation is consistent with Sheng et al.’s (2003) assertion that processing in a NWM task (letter match) requires subjects to compare many features of the letters (e.g., shape, pronunciation, etc.), while a SWM task (location match) requires only a single integrated feature, spatial location, to be noticed (Sheng et al., 2003). This difference may result in a shorter RT in a SWM task than in a NWM task. In an event-related potential study, Mecklinger and Müller (1996) found that the P200 component evoked at posterior electrodes by a NWM task was more positive than that evoked by a SWM task. They also found that the P300 component evoked by a SWM task was 100 ms earlier than that evoked by a NWM task. Wo et al. (2005) found that slow cortical potentials evoked by NWM task performance were significantly more negative in the PFC, and more delayed relative to the stimulus in posterior cortices (~1400 ms vs. ~700 ms), than slow cortical potentials evoked by SWM task performance. These results hint that NWM may involve broader and slower processing than SWM. Although we attempted to reduce phonetic matching by using both uppercase and lowercase letters, the phenomenon may have still occurred.

## Conclusion

Concurrent postural control demands interfere selectively with SWM, but not NWM, performance discernibility and SWM task-related brain activity in frontal-parietal areas. Conversely, SWM processing demands affect posture stability more than NWM processing demands. Furthermore, the multiple linear regression analysis showed that SWM, but not NWM, was an important predictor of postural control. These findings suggest that SWM may share more cognitive resources with postural control than NWM.

## Author Contributions

YL and YC designed experiments. YC, YY, RN and YL conducted experiments and analyzed the data. YC wrote the article.

## Conflict of Interest Statement

The authors declare that the research was conducted in the absence of any commercial or financial relationships that could be construed as a potential conflict of interest.
